# Using Empirical Bayes Methods to Rank Counties on Population Health Measures

**DOI:** 10.5888/PCD10.130028

**Published:** 2013-08-01

**Authors:** Jessica K. Athens, Bridget B. Catlin, Patrick L. Remington, Ronald E. Gangnon

**Affiliations:** Author Affiliations: Bridget B. Catlin, Patrick L. Remington, Ronald E. Gangnon, University of Wisconsin, Madison, Wisconsin.

## Abstract

The University of Wisconsin Population Health Institute has published *County Health Rankings* (*The Rankings*) since 2010. These rankings use population-based data to highlight variation in health and encourage health assessment for all US counties. However, the uncertainty of estimates remains a limitation. We sought to quantify the precision of *The* *Rankings* for selected measures. We developed hierarchical models for 5 health outcome measures and applied empirical Bayes methods to obtain county rank estimates for a composite health outcome measure. We compared results using models with and without demographic fixed effects to determine whether covariates improved rank precision. Counties whose rank had wide confidence intervals had smaller populations or ranked in the middle of all counties for health outcomes. Incorporating covariates in the models produced narrower intervals, but rank estimates remained imprecise for many counties. Local health officials, especially in smaller population and mid-performing communities, should consider these limitations when interpreting the results of *The* *Rankings.*

## Introduction

*County Health Rankings *(*The Rankings*) (www.countyhealthrankings.org), first published in 2010 by the University of Wisconsin Population Health Institute (the Institute), assesses population health for nearly all US counties. *The* *Rankings* is designed to direct media and policy-maker attention toward the multiple determinants of health and encourage in-depth community health assessment (1). Community engagement resulting from *The Rankings* is intended to motivate the implementation of evidence-based programs that address upstream determinants of health.

The Institute uses federal and other national data sources that provide county estimates of health-related measures. Measures from federal data sources are commonly censored if the number of events or the sample size is small, because of privacy concerns; the reported estimates are often imprecise (2). For greater estimate stability, the Institute has aggregated up to 7 years of data for select measures. Nonetheless, small sample sizes and large standard errors have remained common across many measures. We therefore posed the following questions: How precise are the rankings in *The Rankings*, and can we increase the precision of the rankings?

To address these questions, we used hierarchical Bayesian models for the health outcomes data in the 2010 *Rankings*. Hierarchical models identify components of variation at the state and county levels, as well as sampling variation, and naturally impute values for counties without data. In a Bayesian context, the random and fixed effects estimated in our hierarchical models represent underlying probability distributions from which we could sample a set of plausible values (posterior samples) for each county and measure. By using these samples we calculated point estimates and the Bayesian analog to confidence intervals, “credible intervals” (3). Measures were first modeled with state and county random effects only, and then demographic covariates were added to determine whether they improved rank precision.

This work builds on previous efforts using multilevel models to generate small-area estimates (4–8). These earlier efforts, however, used individual-level data that are not readily available to local health officials. This article emphasizes the use of reported data aggregated to the county level in hierarchical models to generate empirical Bayesian point and interval estimates for ranks. The intent is to explore approaches for obtaining more informative and more precise county health rankings*.*

### Technical overview of *The Rankings*

The Institute analyzes data for more than 25 health-related measures in *The* *Rankings*. Five of these measures represent health outcomes or the current health of a community. The remaining 20 health factors measures represent the determinants of health outcomes. Determinants are divided into 4 categories: health care, health behaviors, socioeconomic status, and the physical environment.

Rather than report an individual rank for each measure, *The* *Rankings* calculates and reports in-state county ranks for composite measures, such as health outcomes or health factors, and their subcategories. To calculate a health-outcomes rank, for example, the 5 health-outcomes measures are converted to in-state *z* scores, and the weighted mean of these *z* scores is then ranked (9) (Table 1).

**Table 1:** *County Health Rankings* Weights of Health-Outcome Measures

Measure	Weight **a**
Premature mortality	50%
% Reporting fair or poor health	10%
Mean no. of poor physical health days per month	10%
Mean no. of poor mental health days per month	10%
% Live births with low birth weight (<2,500 g)	20%

a The weights represent the relative contribution of each measure to the composite health outcomes score.

In this article, we restricted our analysis to the 5 health outcome measures. Because measure-based ranks do not align with *The* *Rankings* results, we focused on the performance of the composite health-outcomes rank.

## Methods

### Data

The 2010 *Rankings* uses several national measures and data sources for health outcomes (Table 2). For all measures but premature mortality, the analyses use National Vital Statistics System (NVSS) (http://www.cdc.gov/nchs/nvss.htm) and Behavioral Risk Factor Surveillance System (BRFSS) (http://www.cdc.gov/brfss/)) data aggregated to the county level and reported to the Institute.

**Table 2:** Data Sources for 2010 *County Health Rankings* Health Outcome Measures

Measure	Source	Years
**Premature mortality**		
Premature mortality (years of potential life lost before age 75 per 100,000 population)	National Vital Statistics System, National Center for Health Statistics^a^ — reported in *The* *Rankings*	2004–2006
Raw mortality and population counts by age group	Underlying cause of mortality query, CDC Wide-ranging Online Data for Epidemiologic Research (2) — used in analysis	
% Reporting fair or poor health	Behavioral Risk Factor Surveillance System^b^	2002–2008
Mean no. of poor physical health days per month	Behavioral Risk Factor Surveillance System^b^	2002–2008
Mean no. of poor mental health days per month	Behavioral Risk Factor Surveillance System^b^	2002–2008
% Live births of babies with low birth weight (<2,500 g)	National Vital Statistics System, National Center for Health Statistics^a^	2000–2006

a National Vital Statistics System (http://www.cdc.gov/nchs/nvss.htm).

b Behavioral Risk Factor Surveillance System (http://www.cdc.gov/brfss/).

*The Rankings* represents premature mortality by years of potential life lost before the age of 75 (YPLL-75), where each death in a county is weighted based on the difference between age 75 and age at death (10). Because YPLL-75 rates are a composite of age-specific death rates, we directly modeled the underlying mortality rates for 9 age groups and used the resulting output to calculate posterior samples of YPLL-75. Raw mortality and population counts for age under 1 year, ages 1 through 4 years, and 10-year age groups between ages 5 and 74, were accessed from the Center for Disease Control and Prevention’s Wide-ranging Online Data for Epidemiologic Research (WONDER) underlying cause-of-death query system (2).

County data on race/ethnicity, sex, and age for 2008 were accessed through the US Census Population Estimates program (11). Urbanization classification data are from the National Center for Health Statistics (12).

The percentage of counties with missing health outcome data ranged from 3.1% to 13.7%. Rural counties accounted for the majority of counties with missing data (72%–92%). Values for vital statistics measures were suppressed if based on 5 or fewer events. BRFSS censored values for counties with fewer than 50 respondents or a 95% confidence interval width greater than 20%.

### Hierarchical models

For each health outcome measure, we entered the data into 2 generalized, linear, mixed-effects models with state- and county-level random effects and either an intercept only (model 1) or an intercept plus county-level demographic covariates as fixed effects (model 2). We used the demographic variables to inform estimates, not to adjust for county differences. The Poisson model specifications are below:


*y_jk_* ~ Poisson(*ρ_jk_*_,_
*n_jk_*)

Model 1: log(*ρ_jk_*) = *β_o_* + *e_j_* + *e_jk_*

Model 2: log(*ρ_jk_*) = *β_o_* + *β_1_* (African American) + *β_2_* (Asian) + *β_3_* (American Indian) + *β_4_* (Latino) + *β_5_* (Urbanization) + *β_6_* (Female) + *β_7_* (<Age 18) + *β_8_* (>Age 64) + *e_j_* + *e_jk_*

*e_j_* ~ N(0, *σ_j_^2^*)

*e_jk_* ~ N(0, *σ_jk_^2^*)

Where

*y_jk_* = number of events in county *k*, state *j*

*n_jk_* = population in county *k*, state *j*

*ρ_jk_* = event rate for county *k*, state *j*

*β_o_* = Intercept, or the average log event rate across all counties

*β_1_* …*β_8_* = County-level demographic covariates

*e_j_* = state-specific random effect parameter

*σ_j_^2^* = variance of state-specific random effects

*e_jk_* = county-specific random effect parameter

*σ_jk_^2^* = variance of county-specific random effects

For *j* = 1, 2, …, 51; *k* = 1, 2, …, *m_j_*

#### Poisson models

The mortality rates underlying premature mortality followed a Poisson distribution; data for each age group included number of events and population denominator.

#### Binomial models

We used a binomial distribution to model low birth-weight births and self-reported health. Low birth-weight data included a census of all live births and the number of births of babies with low birth weight. Self-reported health data included a point estimate and 95% confidence limits. The implied standard errors given by the confidence limits and the reported prevalence were used to obtain the effective numerator and denominator for each county.

#### Log-normal models

The measures of poor physical and poor mental health days approximated a Gaussian distribution but were log-transformed to control for over-dispersion. Reported data for these measures included mean value by county, the number of respondents, and 95% confidence limits. We used the inverse of county-specific variances, obtained from the confidence limits, as weights to account for sampling error.

#### Model covariates

Demographic covariates included categorical variables for percentage African American, Asian, American Indian, and Latino; percentage female; percentage under age 18 years and over age 64 years; and urbanization. We chose not to include as covariates any measures we considered modifiable factors, particularly those that comprise the health factors rank, which include variables such as poverty and education. Urbanization was categorized as large metropolitan, small metropolitan, and rural. Race/ethnicity measures were categorized as low (< 6%), medium (6%–39%), and high (≥ 40%) proportion of the population. Covariates for sex and age were divided into 4 categories (Table 3) (13).

**Table 3:** Categorization of Demographic Covariates for Use in Hierarchical Models in Ranking Counties on Population Health Measures

Measure	Category 1 (Low)	Category 2	Category 3	Category 4 (High)
% Female	<45%	45%–50%	50%–55%	>55%
% Younger than 18	<20%	20%–23.5%	23.5%–27%	>27%
% Older than 64	<10%	10%–15%	15%–20%	>20%

### Estimation and fit

Model parameters were estimated by maximum likelihood. Empirical Bayes estimates of the random effects were obtained by conditioning on the estimated variance parameters. Samples of the regression coefficients and state- and county-level random effects were drawn from a multivariate normal approximation to their joint-posterior distributions. Posterior samples of the county-specific estimates were obtained from the sampled vectors. To generate composite scores for ranking, posterior samples for the individual measures were transformed to (national) *z* scores. The measure *z* scores were weighted according to *The* *Rankings* scheme and summed to calculate posterior health outcomes scores. Estimation was performed in R (version 2.14.1, R Foundation for Statistical Computing, Vienna, Austria), by using the lme4 and MASS libraries (14,15).

#### Comparison to reported data

We used random-number generation procedures for binomial, Poisson, and normal distributions to produce posterior-predictive data sets based on the posterior samples and the reported population data for each measure. Similarities among parameters calculated from the posterior-predictive data sets and the original data were quantified by posterior predictive *P* values. These *P* values are similar to traditional *P* values used to assess statistical significance, in which a probability is set at a threshold below which the null hypothesis is rejected. In this case, a significant *P* value suggested the observed data were different from the simulated data, indicating poor fit (16,17). We selected the interquartile range (IQR) as our summary measure of a variable’s distribution.

Posterior samples of county ranks were obtained by ranking the county-specific *z* scores for health outcomes within each sample. Point estimates of the ranks were obtained by ranking the posterior mean ranks. The central 90% of the distribution of the posterior ranks represented the credible (confidence) interval for a county’s rank. We also calculated the posterior probability of a county ranking in its assigned quartile.

## Results

### Estimates

We found that the models for self-reported health, low birth-weight births, and poor mental health days were best able to replicate the IQRs of the original data. The models that fit less well were poor physical health days and premature mortality, for which the observed IQRs fell within the distribution of the IQRs calculated from posterior-predictive samples less than 5% of the time. Overall, the challenges in model fit make our posterior rank estimates less reliable indicators of county performance for these 2 measures.

### National rank certainty

Using data from all US counties allowed us to explore national rank performance. We observed significant clustering by state; this clustering, along with wide 90% credible intervals, made in-state credible intervals impractical and inconsistent with our models. When we used posterior samples from the empty models, the mean width for the 90% credible intervals was 565 ranks (18 percentile ranks) for health outcomes. Adding demographic covariates into the hierarchical models increased rank precision marginally: the mean width of the confidence intervals decreased by 2.7 percentile ranks.

**Figure 1:**
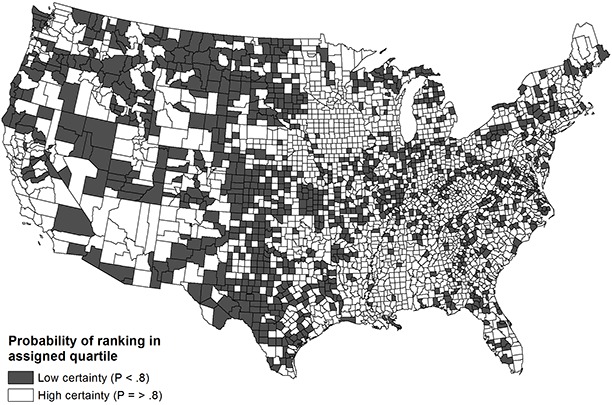
Choropleth map of US county rank certainty in composite health outcomes (empty model).

**Figure 2:**
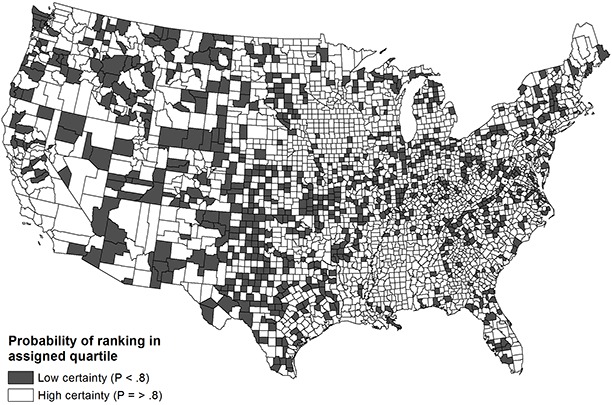
Choropleth map of US county rank certainty in composite health outcomes (demographic model).

The probability of counties ranking in their assigned national quartiles for health outcomes is based on the empty (Figure 1) and demographic (Figure 2) models. The probability is dichotomized into high certainty (*P* ≥ .80) and low certainty (*P* < .80). The maps in the figures demonstrate marginal improvement in rank certainty when using the estimates informed by demographic covariates. Whereas 65% of counties (n = 2,036) ranked in their assigned quartile with high certainty based on the empty model, 73% of counties (n = 2,293) ranked with high certainty using the demographic model. Counties that had wide confidence intervals commonly had small populations and were concentrated in the Mountain and central South regions; these counties also tended to rank in the middle quartiles nationally. Ranks for the components of health outcomes, mortality and morbidity, were less precise than the composite rank.

## Discussion

The estimates for health outcome measures used in *The* *Rankings* were often imprecise, especially for counties that have smaller populations. The empirical Bayes estimates stabilized these values, especially for the outliers for counties that have small populations. Models using state-specific and county variances were also able to impute estimates for counties without data more effectively than models using mean substitution. These features of Bayesian estimates make them ideal for ranking, but they suggest that our estimates of rank certainty overstate the precision of the reported rankings.

Our results are consistent with Hall and Miller’s examination of rank performance, in which a small group of “highly performing” entities tends to remain fixed in rank (18). Adding covariates confirms the rank location of counties at the extremes, but the value-added is limited for middle-ranking counties. The Institute cautions *Rankings* users against over-interpreting their county’s rank, and our results confirm that the performance of counties based on the public data used in *The Rankings* can be uncertain. The wide intervals we observed for many counties make explicit to policymakers and stakeholders the challenges of comparing counties using ranks.

Our primary goal was to use the posterior samples of the county-specific parameters to estimate precision, but these samples can provide other advantages, such as the flexibility to report national performance as well as performance within state. The Institute does not report national ranks for numerous salient reasons, including the importance of focusing media attention on county health in all 50 states. However, reporting national percentile ranks alongside or in place of in-state ranks can provide richer information on how counties perform.

The hierarchical models also allow for the calculation of race-, sex-, and age-adjusted estimates for ranking. In their current configuration, *The* *Rankings* compares diverse communities with significant variation in demographic and structural factors associated with health outcomes. These unadjusted ranks are crucial for identifying communities in need of resources, but are not ideal for assessing performance. Controlling for factors that are strongly correlated with health outcomes at the population level allows for the calculation of risk-adjusted ranks that may more adequately measure county performance (19).

In this study we examined cross-sectional data with a limited set of demographic covariates. These demographic covariates, furthermore, were assumed to be national-level fixed effects as — for many states — there is insufficient data for state-level fixed effects. Future work will explore the use of demographics covariates as state-level random effects to better account for the variable association between race/ethnicity and health outcomes by state.

There are several potential extensions of these hierarchical models to improve point and interval estimation of ranks, including the use of more extensive demographic covariates in the cross-sectional model, the use of longitudinal data on a single health outcome and the use of multiple related outcomes in a single hierarchical model. These expanded models may be particularly useful in addressing challenges with model fit with the poor physical health days and premature mortality measures.

The models provide automatic spatial smoothing of county event rates within states through the inclusion of a state-level random effects component. Although this eliminates the need to specify or estimate a spatial lag parameter, as done elsewhere for states (20) and counties (7,21), the assumed similarity of rates within states may magnify differences among neighboring counties in different states.

Now in its fourth year, *The Rankings* has proven to be a successful motivator of population health improvement in its current format. Because of data and methods challenges, the benefits of *The Rankings* in its current configuration are 1) the presentation of an accessible and comprehensive framework for understanding population health and 2) the use of ranking to draw attention to and motivate improvements in community health. Given their uncertainty, rankings are not presented nor should they be considered as definitive assessments of county health performance. The approach here explores ways to build on the advantages inherent in *The Rankings*, including quantifying and, where possible, improving rank precision. Extensions of these models may further improve rank certainty, as well as help make *The* *Rankings* more useful for assessing performance and targeting resources.
